# Advancing the Role of Gamma-Tocotrienol as Proteasomes Inhibitor: A Quantitative Proteomic Analysis of MDA-MB-231 Human Breast Cancer Cells

**DOI:** 10.3390/biom10010019

**Published:** 2019-12-21

**Authors:** Premdass Ramdas, Ammu Kutty Radhakrishnan, Asmahani Azira Abdu Sani, Mangala Kumari, Jeya Seela Anandha Rao, Puteri Shafinaz Abdul-Rahman

**Affiliations:** 1 Department of Molecular Medicine, Faculty of Medicine, University of Malaya, 50603 Kuala Lumpur, Malaysia; premdass_ramdas@imu.edu.my; 2 Department of Medical Biotechnology, School of Health Sciences, International Medical University, 57000 Kuala Lumpur, Malaysia; 3 Jeffrey Cheah School of Medicine and Health Sciences, Monash University Malaysia, Bandar Sunway, 47500 Selangor, Malaysia; ammu.radhakrishnan@monash.edu; 4 Malaysian Genome Institute, National Institute of Biotechnology, 43000 Bangi, Malaysia; 5 Division of Human Biology, International Medical University, 57000 Kuala Lumpur, Malaysia; mangala_kumari@imu.edu.my; 6 Division of Pathology, International Medical University, 57000 Kuala Lumpur, Malaysia; JeyaSeela@imu.edu.my; 7 University of Malaya Centre of Proteomics Research (UMCPR), University of Malaya, 50603 Kuala Lumpur, Malaysia

**Keywords:** vitamin E, tocotrienols, mass spectrometry, proteasomes, breast cancer

## Abstract

Tocotrienol, an analogue of vitamin E has been known for its numerous health benefits and anti-cancer effects. Of the four isoforms of tocotrienols, gamma-tocotrienol (γT3) has been frequently reported for their superior anti-tumorigenic activity in both in vitro and in vivo studies, when compared to its counterparts. In this study, the effect of γT3 treatment in the cytoplasmic and nuclear fraction of MDA-MB-231 human breast cancer cells were assessed using the label-free quantitative proteomics analysis. The cytoplasmic proteome results revealed the ability of γT3 to inhibit a group of proteasome proteins such as PSMA, PSMB, PSMD, and PSME. The inhibition of proteasome proteins is known to induce apoptosis in cancer cells. As such, the findings from this study suggest γT3 as a potential proteasome inhibitor that can overcome deficiencies in growth-inhibitory or pro-apoptotic molecules in breast cancer cells. The nuclear proteome results revealed the involvement of important nuclear protein complexes which hardwire the anti-tumorigenesis mechanism in breast cancer following γT3 treatment. In conclusion, this study uncovered the advancing roles of γT3 as potential proteasomes inhibitor that can be used for the treatment of breast cancer.

## 1. Introduction

Breast cancer (BC) is the most common malignancy in women around the world. According to GLOBOCAN 2018, breast cancer is the most common cancer in women; accounting for 11.6% of all cancers [1]. In 2018, about 2.1 million newly diagnosed female breast cancer cases were reported worldwide, which accounts for about 1 in 4 breast cancer cases among women [1]. The same authors reported that there were 626,679 (6.6%) cases of deaths due to breast cancer worldwide [1]. Hence, the incidence of breast cancer in women worldwide was reported to be 24.2%, while the mortality rate was estimated to be 15% [1]. A similar trend was observed in the United States of America (USA) where the incidence rate of cancer was reported to have increased over the last decade. In 2018, there was a total of 1,735,350 new cancer cases and 609,640 cancer-related deaths in the USA [2]. These global breast cancer data confirmed that this malignancy is a leading concern across the globe and pose serious threats to the well-being of women worldwide. The rise in the incidences of breast cancer in these countries could be due to adverse life style changes, which increase the risk for this disease. The mortality of breast cancer in these regions is increasing, with a lack of proper diagnosis and therapy being the main reason [3]. The treatment options for a breast cancer patient can vary from one patient to another, depending on the tumor biology and sub-types (e.g., triple negative or HER2-positive). Surgery is still the mainstay of treatment in early breast cancer [4]. Cancer treatment modalities such as chemotherapy, radiotherapy, hormonal therapy, and surgery often cause major side-effects in the patients [5]. Some of the side-effects associated with breast cancer treatment can affect the quality of life of these patients, which can affect every part of their bodies. Furthermore, triple-negative breast cancers do not respond to hormonal therapy [6]. Traditional chemotherapy and radiotherapy can also kill normal healthy cells along with the cancer cells, which causes severe systemic syndrome such as nausea, pain, and lethargy [7]. These situations urge the researchers to find alternative strategies that can be used to effectively prevent and treat breast cancer with negligible side effects. One such a strategy is the use of bioactive natural compounds to combat carcinogenesis, especially those that selectively kill cancer cells while having beneficial effects for sustaining health.

Tocotrienols (T3), a member of the vitamin E family, consist of four naturally occurring isoforms, namely α-, β-, δ-, and γ-tocotrienols. Numerous evidences suggest that tocotrienols are effective anti-cancer bioactive compounds and its selectivity against cancer cells was demonstrated profoundly in both cell-based and animal studies. Studies have shown that tocotrienols were able to induce apoptosis in malignant cells, but not in non-malignant cells [8,9]. This selectivity could be largely due to the reduced antioxidant defense expressed by these malignant cells when compared to non-malignant cells [10] or might involve many other unknown factors. The selectivity of T3 for cancer cells might be dependent on the ability of the former to specifically target and downregulate proteins, which are abnormally upregulated in carcinogenic but not in normal cells. Research on tocotrienols offers a promising discovery as this compound has been reported to combat oncogenesis by targeting multiple cell signaling pathways [11].

One such important pathways that was explored in this study is the ubiquitin–proteasome pathway, which is one of the major regulatory system for intracellular protein degradation [12]. The ubiquitin–proteasome pathway plays a key role in cancer therapy. Proteasome inhibition was also reported to potentiate the anti-cancer efficacy of other chemotherapeutic drugs by decreasing the expression of anti-apoptotic proteins and increasing the levels of pro-apoptotic proteins [13]. Identification of novel compounds that can block the proteolytic activities in cancer cells by inhibiting the proteasomes is a potent strategy for cancer prevention and treatment [14]. As such, this research was embarked to identify differentially expressed proteins in MDA-MB-231 cells exposed to gamma-tocotrienol (γT3) treatment, using label-free quantitative proteomics and functional bioinformatics strategies to get into the novel insights of anti-cancer mechanisms of γT3 and its potential therapeutic strategies for breast cancer.

## 2. Materials and Methods 

### 2.1. Cell Line and Treatment Conditions

The MDA-MB-231 cells were grown in T75 flasks in Dulbecco’s Modified Eagle Medium (DMEM) supplemented with 10% Fetal Bovine Serum (FBS) in a humidified atmosphere of 5% carbon dioxide in air at 37 °C, until it reaches 70–90% confluency. A total of 5 × 10^5^ cells were seeded in T75 flask containing 10 mL of complete media. Following cell adherence, the cells were treated with IC_50_ concentration, 5.8 µg × mL^−1^ of γT3, as established previously [15]. Following 24 h of γT3 treatment, the cells were washed with 1× PBS solution. A cell scrapper was used to dislodge the attached cells. The cells were collected in 5 mL PBS and washed with two volumes of ice-cold PBS and centrifuged at 250 × *g* for 5 min at 4 °C. The supernatant was discarded, and the pellets were resuspended in 40 mL of ice-cold PBS and centrifuged as before. The washing step was repeated twice. The pellet was subjected to two separate processing steps to extract (i) cytoplasmic proteins and (ii) nuclear proteins.

### 2.2. Extraction of Protein Fractions

#### 2.2.1. Isolation of Cytoplasmic Fraction

The cytoplasmic protein fractions were extracted using the CHEMICON^®^’s cytosol protein fraction extraction kit (USA-Catalog No. 2900), according to the manufacturers protocol [16]. In brief, five times the pellet volume of ice-cold 1x cytoplasmic lysis buffer containing 0.5 mM DTT and 1/1,000 dilution of inhibitor cocktail were added to the pellet. The cell pellet was resuspended by gently inverting the tube and was incubated on ice, for 15 min. Following this step, the cell pellet suspension was centrifuged at 250 × *g* for 5 min at 4 °C. The supernatant was discarded, and the cell pellet was resuspended in two volumes of ice-cold 1x cytoplasmic lysis buffer. Using a syringe with a small gauge needle (27 G), the prepared cell suspension was drawn from a sample tube and the contents were ejected back into the sample tube. These steps (drawing and ejecting) were repeated approximately five times. The disrupted cell suspension was centrifuged (8,000 × *g* for 20 min at 4 °C) and the supernatant, which contains the cytosolic portion of the cell lysate was transferred to a fresh tube and was used for cytoplasmic proteome analysis.

#### 2.2.2. Isolation of Nuclear Fraction

The nuclear pellet from the cytoplasmic protein extraction step was resuspended in 2/3 of the original cell pellet volume of ice-cold nuclear extraction buffer containing 0.5 mM DTT and 1/1,000 protease inhibitor cocktail. Using a fresh syringe, with a 27-gauge needle, the pellet was drawn and ejected into the tube to disrupt the nuclei. Using a rotator or orbital shaker (low speed), the nuclear suspension was gently agitated at 4 °C for 30–60 min. The nuclear suspension was then centrifuged at 16,000 × *g* for 5 min at 4 °C. The resulting supernatant, which contained the nuclear proteins, was transferred to a fresh tube and used for nuclear proteomic analysis.

### 2.3. Determination of Protein Concentration and Trypsin Digestion

The concentration of the extracted protein was determined by a bicinchoninic acid (BCA) assay and bovine serum albumin (BSA) was used as a protein standard for the calibration curve. Approximately 100 µg of total protein was suspended in 100 ul of 50 mM ammonium bicarbonate (pH 8.0) containing 0.05% RapiGest (Waters Corporation, Milford, MA). The solution was incubated at 80 °C for 15 min. Protein was reduced in the presence of 5 mM dithiothreitol (DTT) at 60 °C for 30 min. The protein was alkylated in the dark in the presence of 10 mM iodoacetamide at room temperature, for 45 min. Proteolytic digestion was initiated by adding a trypsin gold at a concentration of 100:0.25 (protein to trypsin) and was incubated at 37 °C overnight. Tryptic digestion and RapiGest activity were terminated by adding a 1 µL concentrated trifluoroacetic acid (TFA) incubated at 37 °C for 20 min. The tryptic peptide solution was centrifuged at 14,000 rpm for 20 min and the supernatant was transferred into a clean microcentrifuge tube and kept at −80 °C until further analysis.

### 2.4. Liquid Chromatography and Mass Spectrometry Analysis

Digested peptides were analyzed by The Dionex^TM^ UltimateTM 3000 RSLCnano from Thermo Scientific^TM^. The peptides were desalted and concentrated in a trapping column C_18_ PepMap^TM^ 100, 5µm, 100Å and were separated by Easy-Spray^TM^ LC Column Acclaim PepMap^TM^ C_18_ 75µm id × 25 cm, with a 40% acetonitrile gradient over 120 min, at a flow rate of 300 nL/min. Each sample was injected in triplicates. All spectra were acquired on an Orbitrap Fusion (Thermo Scientific^TM^) in data-dependent mode, with positive ionization. Full scan spectra were collected at a resolution of 120,000, with an automated gain control (AGC) target of 400,000, and the maximum injection time was 50 ms. The method consisted of a 3 s Top Speed Mode where the precursors were selected for a maximum 3 s cycle. Only precursors with an assigned monoisotopic m/z and a charge state of 2–7 were further analyzed for MS/MS. All precursors were filtered using a 20 s dynamic exclusion window and an intensity threshold of 5,000. The MS2 spectra were analyzed at a resolution of 60,000, with an AGC target of 100 and a maximum injection time of 250 ms. Precursors were fragmented by high-energy collision dissociation (HCD) and collision-induced dissociation (CID) at a normalized collision energy (NCE) of 28% and 30%.

### 2.5. Protein Identification and Label-Free Quantification

Data generated were processed using the Thermo Scientific^TM^ Proteome Discoverer^TM^ Software v2.1 with the SEQUEST^®^ HT search engine. The MS ion intensities were calculated based on the accurate mass and time tag strategy. The accurate alignment of the detected LC retention time and the *m*/*z* value across different analyses and the area under the chromatographic elution profiles of the identified peptides could be compared among different samples. For protein identification, data were searched against a Uniprot^®^ human (homo sapiens) database with a 1% FDR criteria using a Percolator^®^. Search parameters were set up to two missed cleavage with fixed modification of carbamidomethylation and variable modification of methionine oxidation, asparagine, and glutamine deamidation. A fragment tolerance of 0.6 Da and a precursor tolerance of 10 ppm were used with trypsin as a digestion enzyme. Identified protein with a SEQUEST^®^ HT score more than 200 or an above 25% sequence coverage or one that had at least two unique peptides, implied a greater confidence in protein identity.

### 2.6. Data Acquisition and Statistical Analysis

Statistical analyses were performed using a Perseus Software v1.5.3.1 (Max Planck Institute of Biochemistry). Each control and treatment samples consisted of three biological replicates and each biological replicate were injected three times to the LC–MS/MS. Protein file with three technical replicates in text format (.txt) from Proteome Discoverer^TM^ were uploaded to the Perseus. The data were log2-transformed to stabilize the variance and scale normalized to the same mean intensity across the technical replicates. The mean for the three biological replicates from the same samples were grouped together in the same matrix and filtered for the valid values of at least two, to eliminate the protein that only presented in one biological replicate. Finally, all biological replicates from all samples were grouped under the same matrix and the missing values in the data were imputed with the random number that are drawn from a normal distribution. Normally the missing values represent a low abundance measurement. The histogram was plotted to get an impression of whether the ratio distributions are similar for all samples or not. Differently expressed protein between control and treatment were detected using a *t*-test. The *p*-value was adjusted for multiple-testing using the Benjamin–Hochberg false discovery rate. The Benjamin–Hochberg test was used as it is one of the powerful procedures that decreases the false discovery rate. Proteins were considered significant and differentially expressed between the two conditions, with an adjusted *p*-value < 0.05 and a *t*-test difference ≤ −1 or ≥ 1 (= 2-fold change).

### 2.7. Bioinformatics and Functional Analysis

#### 2.7.1. Venn Diagram Analysis

The cytoplasmic and nuclear protein data sets were further analyzed using the Venn diagram analysis [17] to identify unique and overlapping proteins between the differentially expressed cytoplasmic and nuclear proteins, following exposure to γT3. The unique and overlapping differentially expressed cytoplasmic and nuclear proteins of γT3-treated MDA-MB-231 cells generated from this analysis were used to explicate related information that could be associated with the biological significance of these proteins.

#### 2.7.2. Protein Set Enrichment Analysis

The DAVID (**D**atabase for **A**nnotation, **V**isualization, and **I**ntegrated **D**iscovery) bioinformatics resources [18,19] were used to perform enrichment analysis for the differentially expressed proteins. The Uniprot ID of differentially expressed proteins were uploaded into the DAVID bioinformatics resources. Enrichment terms were associated with the differential protein list and the total number of proteins involved in each term were generated along with the percentage of protein involved. Modified Fisher Exact *p*-value (EASE score) were generated for enrichment analysis. The output from the analysis were used to mine biologically meaningful information, based on their molecular functions and disease bio-pathways curated from KEGG [20,21] databases. The KEGG is an encyclopedia of genes and genomes used to assign functional meanings to gene/protein elements, both at the molecular and higher levels. The differentially expressed cytoplasmic and nuclear proteins were matched with KEGG pathway databases to generate predicted pathways.

#### 2.7.3. STRING Protein–Protein Interaction Analysis

The functional classification and pathway analysis of significantly different proteins obtained for supernatant, nuclear, and cytoplasmic proteome were performed using the STRING (**S**earch **T**ool for the **R**etrieval of **IN**teracting **G**enes/Proteins) functional protein association network database [22]. The STRING database and its online resources were used to predict functional interactions between differentially expressed proteins, based on its physical binding and regulatory interactions. The protein–protein interactions were analyzed by uploading the uniprot IDs into the multiple protein analysis with Homo Sapiens selected from the organism drop-down option. The protein–protein interactions were assessed using network edges of evidence, confidence, and molecular action. The interactions were generated based on sources from text mining, experiments, databases, co-expression, neighborhood, gene fusion, and co-occurrence. The interaction score was set to high confidence (0.700) and the K-mean clustering were applied to the analysis to populate protein groups with similar interactions.

## 3. Results

### 3.1. Label-Free Mass Spectrometry Quantification of Cytoplasmic and Nuclear Proteins Isolated from MDA-MB-231 Cells following Treatment with γT3

Label-free quantification using the Orbitrap Fusion LC–MS/MS (Thermo ScientificTM) was able to identify cytoplasmic and nuclear proteins that are differentially expressed between γT3-treated and untreated conditions. The resulting proteomic dataset that constituted about 600 proteins were further filtered by fold-change differences, significant *p*-value and false discovery rate (FDR) adjusted *p*-value. From the MDA-MB-231 cells cytoplasmic fractions, a total of 112 proteins were identified to be differentially expressed in response to the γT3 treatment, when compared with the untreated control (Table 1). Among these differentially expressed cytoplasmic proteins, 60 proteins were up-regulated, and 52 proteins were down-regulated. As from the MDA-MB-231 cell nuclear fractions, a total of 79 proteins were identified to be differentially expressed in response to the γT3 treatment, when compared with the untreated control (Table 2). Among these differentially expressed nuclear proteins, 52 were upregulated and 27 were down-regulated by the treatment.

### 3.2. Venn Diagram Analysis

**Figure 1:**
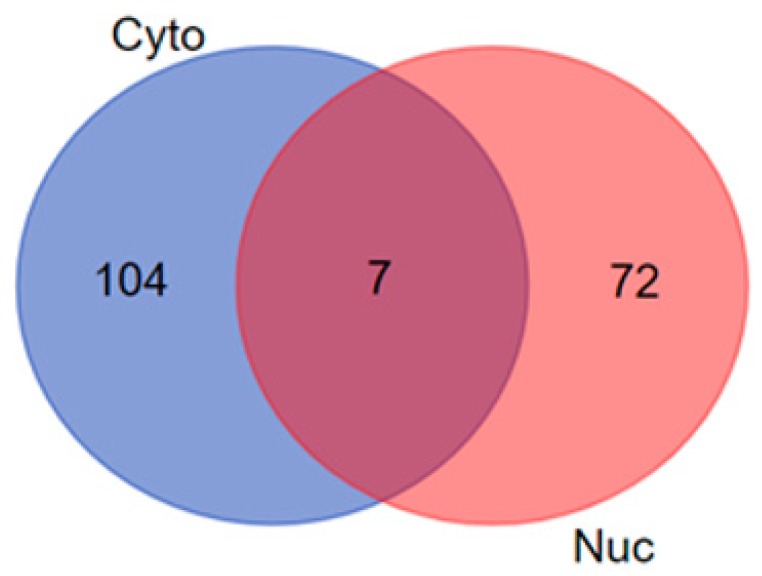
The Venn diagrams shows the intersection of differentially expressed cytoplasmic and nuclear proteins of the γT3-treated MDA-MB-231 cells (cytoplasmic and nuclear compartment). * Cyto: Cytoplasmic; Nuc: Nuclear.

The cytoplasmic and nuclear protein data sets were further analyzed using Venn diagram analysis to identify unique and overlapping proteins between the differentially expressed nuclear and cytoplasmic proteins, following exposure to the γT3. This analysis identified the overlapping regions that illustrates the relations among the differentially expressed cytoplasmic and nuclear protein sets to define the areas of commonality among these two compartments. The Venn diagram analysis revealed a total 104 differentially expressed cytoplasmic and 72 differentially expressed nuclear proteins, respectively. There was a total of seven differentially expressed proteins that were common to both cytoplasmic and nuclear compartments (Figure 1). The list of the unique differentially expressed proteins in the union and intersections between different cytoplasmic and nuclear compartments of MDA-MB-231 information might be associated with the biological significance of these proteins and are shown in Table 3.

### 3.3. Functional Annotation and Pathway Enrichment of Differentially Expressed Proteins of Cytoplasmic and Nuclear Compartment

**Figure 2:**
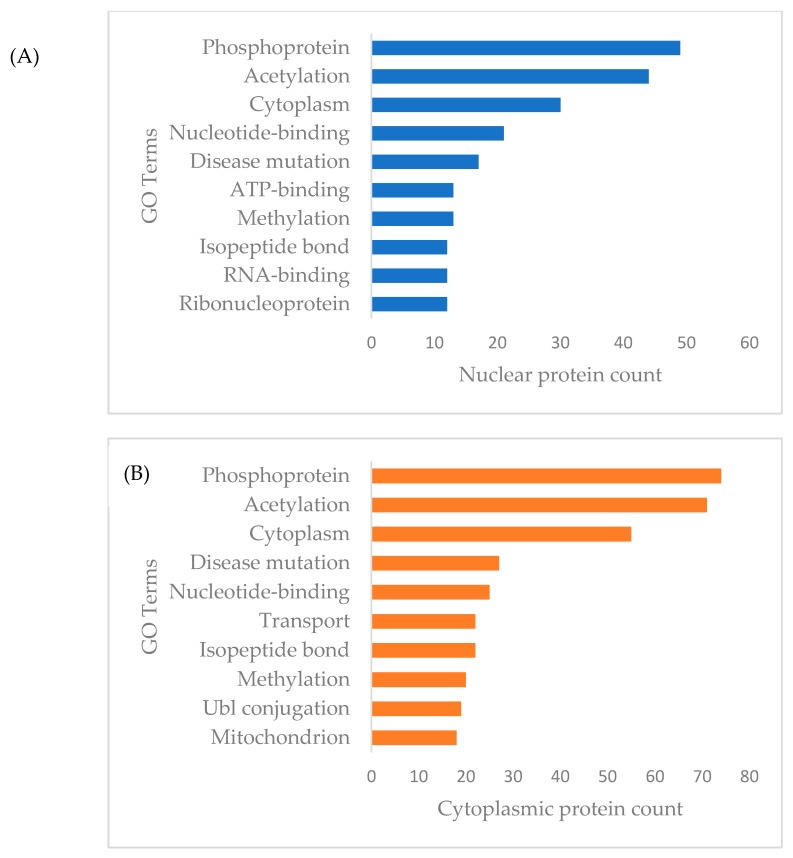
List of differentially expressed (**A**) nuclear and (**B**) cytoplasmic proteins by the γT3-treated MDA-MB-231 cells matched with the functional categories using DAVID analysis. The data showing the top 10 Gene ontology terms for the proteins.

**Figure 3:**
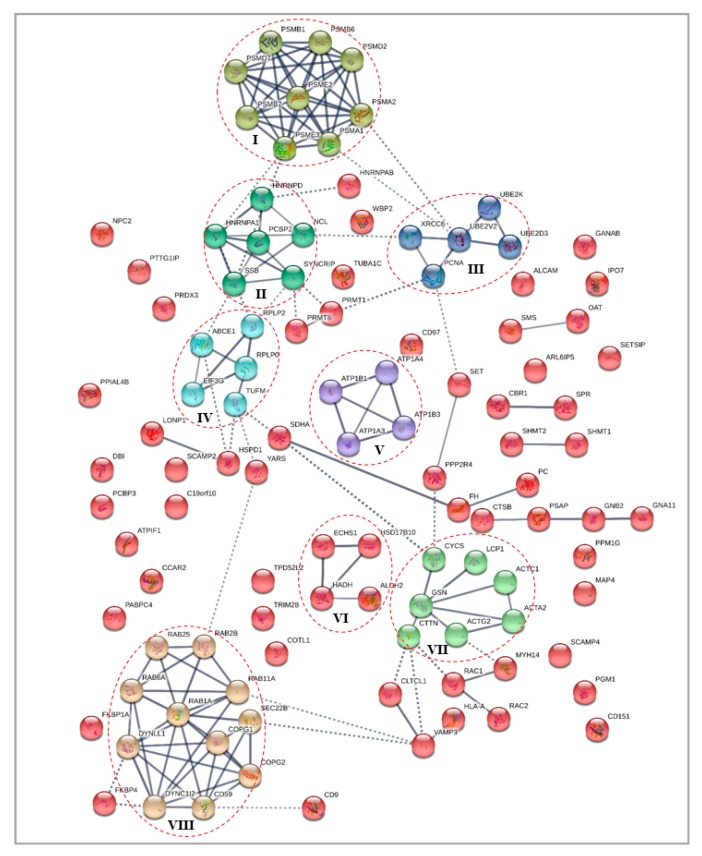
Interaction network for differentially expressed cytoplasmic proteins of MDA-MB-231 cells in response to the γT3 treatment generated using the STRING database. Note: Red dotted circle shows protein clusters with high confidence interactions for each cluster (line thickness indicates the strength of data support).

For the functional categories of differentially expressed cytoplasmic and nuclear proteins of MDA-MB-231 cells in response to γT3 treatment, the analysis revealed that most of these proteins were grouped under phosphoprotein, acetylation, and cytoplasm (Figure 2). Interestingly, there were a total of 74 differently expressed cytoplasmic proteins of MDA-MB-231 categorized as phosphoproteins, based on the DAVID analysis and these proteins might mediate γT3 induced post-translational modification. As for the functional categories of differentially expressed MDA-MB-231 nuclear proteins in response to the γT3 treatment, the analysis showed that most of these proteins are grouped under acetylation, nucleotide binding, ribonucleic, and transport proteins, which confirmed the nature of nuclear fraction used in this study (Figure 3). Interestingly, there were a total of 49 differentially expressed nuclear proteins of MDA-MB-231 categorized as phosphoproteins, based on the DAVID analysis and these proteins might mediate γT3 induced post-translational modification.

### 3.4. Protein–Protein Interaction Analysis

Protein–protein interaction (PPI) analysis were performed to understand the interactions between the key proteins of the differentially expressed cytoplasmic and nuclear fractions, in response to treatment with γT3 in MDA-MB-231 cell lines. The STRING database (http://www.string-db.org/) was utilized to select the interacting protein clusters. In this network, the nodes represent proteins, and the edges represent the interactions between the two proteins, whereby the line thickness between two nodes indicates the strength of data support. The PPI analysis for all protein datasets generated networks with significant interactions than expected, which meant that the differentially expressed proteins had more interactions among themselves than what would be expected for a random set of proteins of similar size, drawn from the genome. Such an enrichment indicated that the proteins are biologically connected as a group/cluster. For all PPI networks, the minimum required interaction score was set at high confidence (0.700) and the K-means clustering were performed to cluster the network to a specified number of clusters, to identify significant protein–protein interaction clusters formed among differentially expressed proteins.

#### 3.4.1. Differentially Expressed Cytoplasmic Proteins STRING Analysis

For differentially expressed cytoplasmic proteins of MDA-MB-231 cells in response to γT3 treatment, the PPI network generated a total of 154 edges, among which 98 edges with high confidence generated a significant (*p* < 9.84 × 10^−8^) PPI enrichment clusters with a local clustering coefficient of 0.466 (Figure 3). A total of eight clusters were generated using K-means clustering, in which prominent interactions of cluster I was formed with PSMB1, PSMB6, PSMD2, PSMA2, PSMA1, PSME3, PSMB7, PSMD7, and PSME3; cluster II with HNRNPD, HNRNPA1, SSB, SYNCRIP, NCL, and PCBP2; cluster III with XRCC6, PCNA, UBE2V2, UBE2K, and UBE2D3; cluster IV with EIF3G, TUFM, RPLP0, RPLP2, and ABCE1; cluster V with ATP1B1, ATP1A3, ATP1B3, and ATP1B3; cluster VI with ECHS1, HSD17B10, HADH, and ALDH2; cluster VII with CYCS, LCP1, ACTC1, ACTA2, ACTG2, CTTN, and GSN; and cluster VIII with RAB25, RAB2B, RAB11A, SEC22B, COPG1, COPG2, CD59, DYNC1I2, DYNLL1, RAB1A, and RAB6A.

#### 3.4.2. Differentially Expressed Nuclear Proteins STRING Analysis

**Figure 4:**
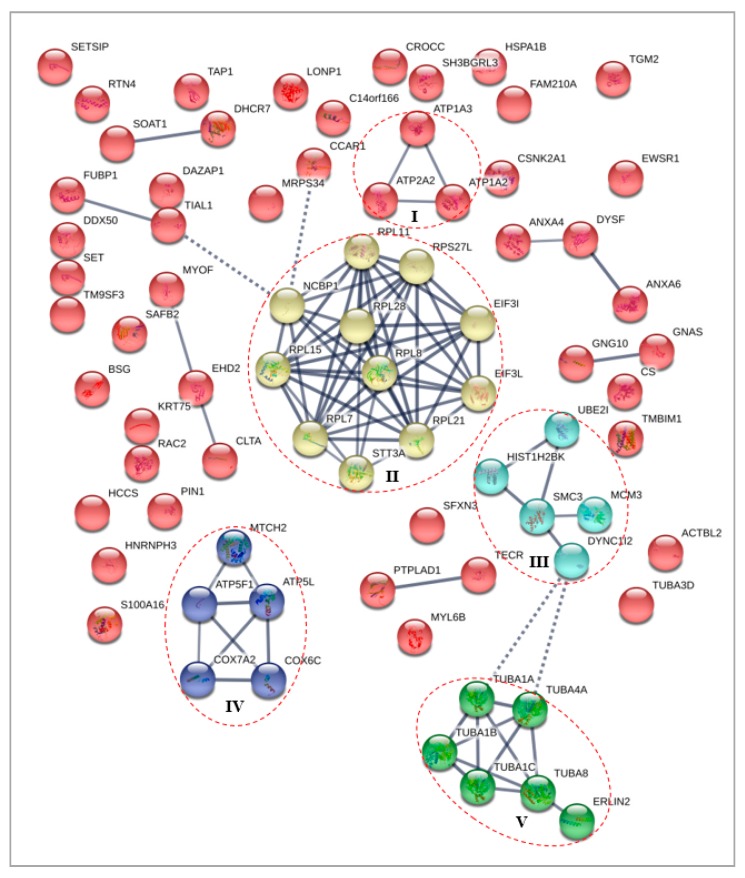
Interaction network for differentially expressed nuclear proteins of MDA-MB-231 cells in response to γT3 treatment generated using the STRING database. Note: Red dotted circle shows protein clusters with high confidence interactions for each cluster (line thickness indicates the strength of data support).

For differentially expressed nuclear proteins in MDA-MB-231 cells in response to the γT3 treatment, the PPI network generated a total of 87 edges, among which 36 edges with high confidence were generated as significant (*p* < 2.3 × 10^−13^) PPI enrichment clusters with a local clustering coefficient of 0.494 (Figure 4). A total of five clusters were generated using K-means clustering, in which prominent interactions of cluster I was formed with ATP1A3, ATP1A2, and ATP2A2; cluster II with RPL11, RPS27L, EIF3I, EIF3L, RPL21, STT3A, RPL7, RPL15, NCBP1, RPL28, and RPL8; cluster III with UBE2I, MCM3, DYNC1I2, TECR, HIST1H2BK, and SMC3; cluster IV with MTCH2, ATP5L, COX6C, COX7A2, and ATP5F1; and cluster V with TUBA1A, TUBA4A, TUBA1B, TUBA1C, TUBA8, and ERLIN2.

## 4. Discussion

**Figure 5:**
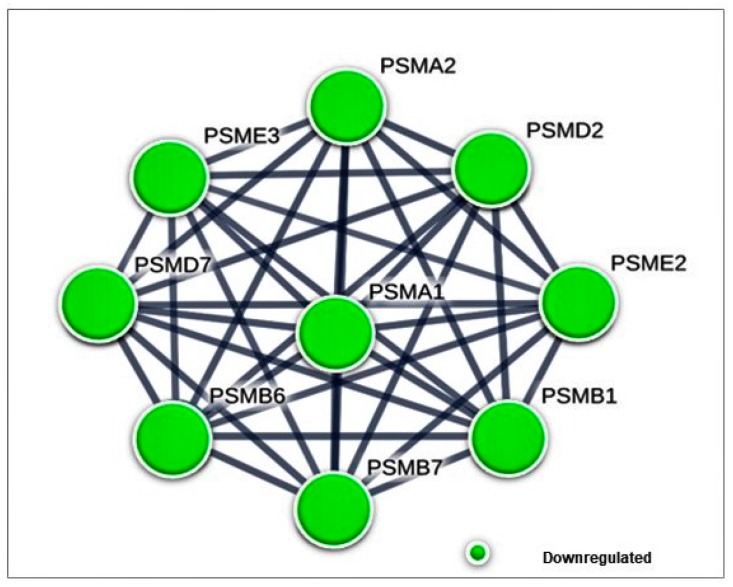
The down-regulated proteasome proteins formed the most prominent network based on the protein–protein interaction populated using the STRING database for differentially expressed cytoplasmic proteins in response to the γT3 in MDA-MB-231 cells.

In this study, differentially expressed cytoplasmic and nuclear proteins of MDA-MB-231 in response to the γT3 treatment were assessed to identify the molecular mechanism through which this isoform of tocotrienol modulates anti-cancer effects. According to the protein–protein interaction (PPI), a total of nine proteasome complex proteins (PSMA1, PSMA2, PSMB1, PSMB6, PSMB7, PSMD2, PSMD7, PSME2, and PSME3) were found to form the most prominent network cluster of differentially expressed proteins in the cytoplasm of the γT3 treated MDA-MB-231 cells (Figure 2). All nine proteins were found to be downregulated by the γT3 treatment. This network of proteins might mediate the proteasome pathway through which γT3 inhibits the proliferation of cancer cells. It has been shown that both transformed and normal cells depend on the function of the proteasome to control the expression of proteins linked to cell survival and proliferation [23]. The proteasome is known to be involved in many distinct regulatory mechanisms in central cellular systems, such as proliferation and apoptosis, and serves as the machinery of proteolysis. Previous study has reported high levels of proteasome activity in breast cancer [24]. This elevated level of proteasome activity is crucial for the survival of cancer cells, as it allows them to escape from the apoptosis mechanism and degrade pro-apoptotic molecules. Cancer cells are more sensitive to proteasome inhibition than normal cells as a reduction in proteasome activity induces apoptosis in these cells [25]. Proteasome activator subunit-3 (PSME3), a protein encoded by the *PSME3* gene, was reported to be upregulated in breast cancer and it promotes protein proteolysis (Figure 5). Knockdown of the *PSME3* gene in human breast cancer cells, suppressed the proliferation of these cells and induced apoptosis [26]. In an experimental model, it was shown that a knockdown of the *PSME3* gene reduced subcutaneous tumor growth rate and increased the number of CD8^+^ T-cells [27]. The PSME protein could also stimulate epithelial mesenchymal transition (EMT) by inducing the expression of cancer stem cell markers, as well as influencing the tumor immune microenvironment by regulating the cell cycle and proliferation of breast cancer cells [27]. In addition, abnormally high expression of PSME3 protein was reported in breast cancer metastatic lymph nodes [28]. It should also be noted that a higher expression of PSME3 had been observed in several types of human cancers and this was found to be a marker of prognosis in breast cancer [29].

**Figure 6:**
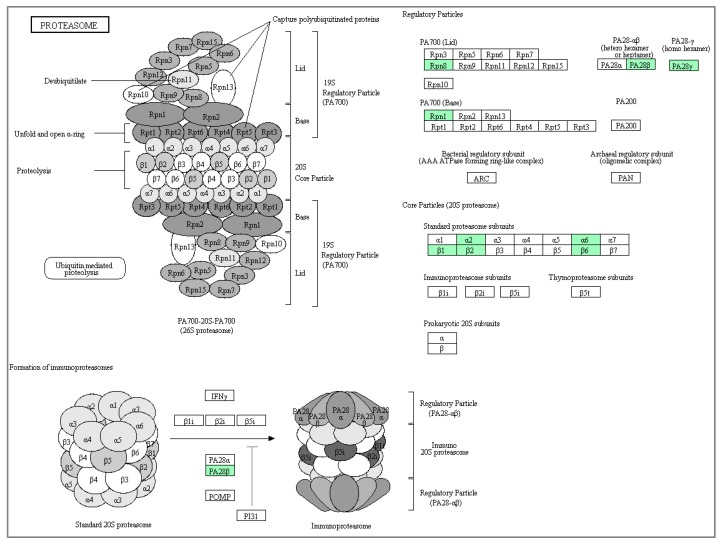
The KEGG proteasome pathway shows involvement of downregulated cytoplasmic. proteins in response to γT3 (green colored boxes) in MDA-MB-231 cells linking the functional role of these proteins to negatively regulate cell proliferation and invasion. (20S proteasome: α2/PSMA2, α6/PSMA1, β1/Psmb6, β2/PSMB7, and β6/PSMB1; regulatory particles: PA28β/PSME2, PA28γ/PSME3; 19S regulatory complex PA700: RPN8/PSMD7, RPN1/PSMD2. (Adapted from KEGG Proteasome Pathway-ko03050 [35]).

Another key protein from this network is the 26S proteasome non-ATPase regulatory subunit-2 (PSMD2). This is a multi-protein complex that is involved in the ATP-dependent degradation of ubiquitinated proteins. Previous study have showed that PSMD2 regulated the cell proliferation and the cell cycle progression in breast cancer via modulation of p21 and p27 proteasomal degradation, which suggest that this protein might be a potential therapeutic target [30]. Analysis using the MALDI-TOF mass spectrophotometry (MS) based on two-dimensional polyacrylamide gel electrophoresis (2-DE) of breast cancer tissues showed over-expression of these proteasomes subunit proteins, including PSMD2 and PSMA1, when compared to normal adjacent tissue, which enhanced the action of ubiquitin–proteasome pathway in breast cancer tissues [31]. Analysis of thousands of tumor samples from The Cancer Genome Atlas (TCGA) reported an increased expression of proteasomes such as PSMB6, PSMB7 [32], and PSMD2 [30] in most cancer types. Other members of the proteasomal protein of this network such as the PSMB7 was reported to be over-expressed in colorectal cancer [33], whilst PSME2 along with PSME3 were reported to be significantly enriched in several biological processes and pathways including cell adhesion, adherent junction organization, regulation of autophagy, cellular protein localization, the cell cycle, and the apoptosis pathway [34]. The ability of the γT3 to downregulate the expression of these proteins delineate tocotrienols as a promising proteasome inhibitor. The crosstalk between these proteins were further investigated using the Kyoto Encyclopedia of Genes and Genomes (KEGG) pathway enrichment analysis. The pathway enrichment predicted the proteasome, as well as inflammation mediated by chemokine and cytokine signaling pathways. In the KEGG proteasome pathway, all nine downregulated proteasome proteins in response to γT3 were seen to be involved in regulating the cellular functions in this pathway (Figure 6). Down-regulation of the proteasome proteins by γT3 in this study shows the ability of this compound to inhibit proteasomes-mediated proteolysis in breast cancer cells. In this pathway, a total of five proteins from the PPI network (Figure 3) are seen to be part of the 20S proteasome core particle, such as α2/PSMA2, α6/PSMA1, β1/PSMB6, β2/PSMB7, and β6/PSMB1, which might regulate their activities in various ways. The immunoproteasome protein Pa28β/PSME2 from the PPI network were represented as one of the two 11S regulatory particles besides PA28α. Other proteins from the PPI network represented as the regulatory particles in the KEGG proteasome pathway are PA28γ/PSME3 and PA700 regulatory particles, RPN1/PSMD2 and RPN8/PSMD7. While proteasome inhibition might have therapeutic benefits to breast cancer therapy, these results represent a novel approach to delineate γT3 as a proteasome inhibitor, which might lead to the development of a new treatment strategy of tocotrienols for breast cancer, as well as others cancer types.

Numerous differentially expressed nuclear proteins were identified in response to the γT3 treatment and were found to be related to important biological processes, such as binding, molecular function regulators, catalytic activities, and translation regulator activities. It is noteworthy that some proteins were known to play important roles in impairing the carcinogenesis. Two upregulated proteins, such as annexin a6 (ANXA6) and scaffold attachment factor B2 (SAFB2), and two downregulated proteins, such as glutamine gamma-glutamyltransferase 2 (TGM2) and S100 calcium binding protein A16 (S100a16), were chosen for discussion, because they were found to be involved directly in the development of carcinogenesis while being found to be dysregulated in response to the γT3 treatment in this study.

Previous studies have showed a low expression of annexin a6 in many cancer types. Low expressions of AXNA6 was reported in melanoma malignancy [36] and epithelial carcinoma, whereby, the overexpression of ANXA6 leads to tumor suppression effects in A431 cells [37]. In breast cancer, low expression of AXNA6 were detected in invasive ductal carcinoma and mucous adenocarcinoma tissues [38] and was predicted to be an important patient survival marker. The AXNA6 also reported to play a potential tumor suppressor role in gastric cancer and have been shown to be down-regulated via promoter methylation in gastric cancer [39]. Recent studies reported the detection of the ANX6 that was found to be enriched in the circulating extracellular vesicles of breast cancer patients undergoing neoadjuvant chemotherapy [40]. Another study also reported that lapatinib, an anti-cancer drug developed by GlaxoSmithKline (GSK), which is used as a treatment for solid tumors such as breast and lung cancer was found to induce ANXA6 expression [41]. In this study, the AXNA6 was upregulated by γT3, implicating the ability of this isoform to mimic the activity of lapatinib.

Previous study suggested the scaffold attachment factor B2 (SAFB2) protein as a tumor suppressor involved in breast cancer development [42]. SAFB2 have also been implicated in breast tumorigenesis and have been reported to be frequently lost in breast cancer due to mutation, while its overexpression results in breast cancer growth inhibition. The same study also reported that in breast cancer patients, low SAFB2 levels are associated with worse outcome in breast cancer patients [43]. Interestingly, in this study the expression of this protein was upregulated by γT3 treatment, which implies that this compound has potential clinical importance in improving the outcome in breast cancer patients.

The protein, glutamine gamma-glutamyltransferase 2 (TGM2) was reported to be downregulated, following treatment with T3 in this study. A higher proportion of mammary tumors showed to express transglutaminase 2 in the chemoprevention group, while its upregulated expression is suggestive of an increased aggressiveness of tumors [44]. Recent studies showed that the TGM2 causes depletion of the P53 tumor suppressor through autophagy in renal cell carcinoma [45] and induces epithelial-to-mesenchymal transition (EMT) in various tumors [46]. Another study on gastric cancer (GC) also suggest that TGM2 might provide a new target for the diagnosis and treatment of GC [47]. Interestingly, the TGM2 protein and mRNA levels were both reported to be elevated in metastases from breast and melanoma cancers [48]. In a nutshell, TGM2 might provide a new target for the diagnosis and treatment of breast cancer, as substantial evidence have shown that down-regulated TGM2 expression can decrease the invasive ability and metastatic potential of breast cancer. The ability of γT3 to downregulate this protein showed a potential therapeutic advantage as a promising natural anti-cancer drug agent in cancer.

Another important down-regulated protein, S100 calcium binding protein a16 (S100A16) has been linked to cancer types including breast cancer with substantial supporting evidences showing its involvement in carcinogenesis. The co-expression of S100A14 and S100A16 was associated with a poor prognosis in human breast cancer as it was reported to promote cancer cell invasion via an interaction with cytoskeletal dynamics [49]. A recent evidence showed that the expression of S100A16 was negatively correlated with the overall survival of bladder cancer patients [50]. Besides breast cancer, S100A16 was identified as an important prognostic marker for colorectal cancer [51] and was shown to be significantly overexpressed in both prostate cancer tissues and cells lines, compared to normal controls [52]. The S100A16 was also upregulated in various types of cancer, including bladder, lung, and pancreatic [53]. While in this study the expression of this protein was significantly down-regulated, previous study have shown that the upregulation of S100A16 expression promotes epithelial mesenchymal transition via the Notch1 pathway in breast cancer [49]. This indicates that γT3 has a potential effect on S100A16, which might negatively regulate some embryonic transcription factors that promotes EMT in breast cancer cells, which are known to be an important target site for the therapy of breast cancer.

## 5. Conclusions

In summary, our data indicates that gamma-tocotrienol (γT3) is a novel proteasome inhibitor adding to its anti-cancer properties. This was the first label-free quantitative proteomics-based study demonstrating the cytoplasmic and nuclear proteins signaling events that were altered in response to the γT3 treatment in MDA-MB-231 cells. This study showed the importance of γT3 as an anti-cancer agent that could be used to replace or support current chemotherapeutic treatment regimes. It might even be useful in devising chemoprevention measures. However, this study had some potential limitations. From the present study, an over-whelming number of differentially expressed proteins in nuclear and cytoplasmic compartments of the MDA-MB-231 human breast cancer cells were identified in response to the γT3 treatment. The roles of all these proteins were inexplicable as it required supporting evidences for further interpretation. Interestingly, some of these proteins have not been previously reported to be modulated by γT3. As such, there is room for further and more in-depth analysis of these data, to elucidate how tocotrienols impair carcinogenesis through dysregulation of various other proteins.

## Figures and Tables

**Table 1:** Differentially expressed cytoplasmic proteins (*p* < 0.05) in γT3-treated MDA-MB-231 cells compared with the untreated control.

**(A) Up-Regulated Cytoplasmic Proteins**			
**Accession**	**Protein Description**	**Gene Symbol**	* Fold-Change
P10809	60 kDa heat shock protein, mitochondrial protein	*HSPD1*	1.45
Q9BQE3	Tubulin alpha-1C chain	*TUBA1C*	2.98
P68032	Actin, alpha cardiac muscle 1	*ACTC1*	1.81
P63267	Actin, gamma-enteric smooth muscle	*ACTG2*	1.81
P62736	Actin, aortic smooth muscle	*ACTA2*	1.81
P62820	Ras-related protein Rab-1A	*RAB1A*	1.71
P30048	Thioredoxin-dependent peroxide reductase	*PRDX3*	1.64
P30084	Enoyl-CoA hydratase, mitochondrial	*ECHS1*	2.15
P34897	Serine hydroxymethyltransferase, mitochondrial	*SHMT2*	1.36
Q99714	3-hydroxyacyl-CoA dehydrogenase type-2	*HSD17B10*	1.42
P48960	CD97 antigen	*CD97*	1.36
P13637	Sodium/potassium-transporting ATPase subunit α-3	*ATP1A3*	1.2
P62879	Guanine nucleotide-binding protein G(I)/G(S)/G(T) subunit beta-2	*GNB2*	2.16
P04181	Ornithine aminotransferase, mitochondrial	*OAT*	2.47
P36776	Lon protease homolog, mitochondrial	*LONP1*	1.45
P05091	Aldehyde dehydrogenase, mitochondrial	*ALDH2*	1.97
Q15366	Poly(rC)-binding protein 2	*PCBP2*	2.13
Q14697	Neutral alpha-glucosidase AB	*GANAB*	2.27
P16152	Carbonyl reductase [NADPH] 1	*CBR1*	1.43
P06396	Gelsolin	*GSN*	1.18
P07954	Fumarate hydratase, mitochondrial	*FH*	1.46
P31040	Succinate dehydrogenase [ubiquinone] flavoprotein subunit	*SDHA*	1.49
P11498	Pyruvate carboxylase, mitochondrial	*PC*	1.12
P07602	Prosaposin	*PSAP*	1.85
P49411	Elongation factor Tu, mitochondrial	*TUFM*	1.7
Q09160	HLA class I histocompatibility antigen	*HLA-A*	3.62
P62942	Peptidyl-prolyl cis-trans isomerase	*FKBP1A*	3.02
Q13733	Sodium/potassium-transporting ATPase subunit α-4	*ATP1A4*	2.58
P04439	HLA class I histocompatibility antigen	*HLA-A*	3.36
O43399	Tumor protein D54	*TPD52L2*	1.68
O75396	Vesicle-trafficking protein SEC22b	*SEC22B*	1.12
Q8WUD1	Ras-related protein Rab-2B	*RAB2B*	2.17
P57721	Poly(rC)-binding protein 3	*PCBP3*	2.31
Q14247	Src substrate cortactin	*CTTN*	1.61
P20340	Ras-related protein Rab-6A	*RAB6A*	1.92
Q15836	Vesicle-associated membrane protein 3	*VAMP3*	2.19
Q7Z406	Myosin-14	*MYH14*	2.33
P21926	CD9 antigen	*CD9*	3.74
P05026	Sodium/potassium-transporting ATPase subunit β-1	*ATP1B1*	1.74
Q14019	Coactosin-like protein	*COTL1*	3.35
P62491	Ras-related protein Rab-11A	*RAB11A*	3.44
Q13740	CD166 antigen	*ALCAM*	1.26
P63000	Ras-related C3 botulinum toxin substrate 1	*RAC1*	1.62
P15153	Ras-related C3 botulinum toxin substrate 2	*RAC2*	2.41
P53801	Pituitary tumor-transforming gene 1 protein-interacting protein	*PTTG1IP*	3.76
Q9Y536	Peptidyl-prolyl cis-trans isomerase A-like 4A	*PPIAL4A*	1.15
P61916	NPC intracellular cholesterol transporter 2	*NPC2*	2.8
P54709	Sodium/potassium-transporting ATPase subunit β-3	*ATP1B3*	2.65
Q969H8	Myeloid-derived growth factor	*MYDGF*	2.75
P63167	Dynein light chain 1, cytoplasmic	*DYNLL1*	1.85
O15127	Secretory carrier-associated membrane protein 2	*SCAMP2*	1.78
Q15819	Ubiquitin-conjugating enzyme E2 variant 2	*UBE2V2*	1.72
P13987	CD59 glycoprotein	*CD59*	1.84
P07108	Acyl-CoA-binding protein	*DBI*	2.14
Q5JXB2	Putative ubiquitin-conjugating enzyme E2 N-like	*UBE2NL*	1.96
P34896	Serine hydroxymethyltransferase, cytosolic	*SHMT1*	2.59
Q969E2	Secretory carrier-associated membrane protein 4	*SCAMP4*	1.68
P48509	CD151 antigen	*CD151*	1.78
P57735	Ras-related protein Rab-25	*RAB25*	2.33
P99999	Cytochrome c	*CYCS*	1.79
**(B) Down-Regulated Cytoplasmic Proteins**			
**Accession**	**Protein Description**	**Gene Symbol**	* Fold-Change
Q99873	Protein arginine N-methyltransferase 1	*PRMT1*	-1.23
P12956	X-ray repair cross-complementing protein 6	*XRCC6*	-1.57
Q01105	Protein SET	*SET*	-1.62
P12004	Proliferating cell nuclear antigen	*PCNA*	-1.46
P19338	Nucleolin	*NCL*	-1.51
Q13200	26S proteasome non-ATPase regulatory subunit 2	*PSMD2*	-1.08
Q9Y678	Coatomer subunit gamma-1	*COPG1*	-2.17
Q9UL46	Proteasome activator complex subunit 2	*PSME2*	-1.63
Q13263	Transcription intermediary factor 1-β	*TRIM28*	-1.82
P05387	60S acidic ribosomal protein P2	*RPLP2*	-1.06
P20618	Proteasome subunit beta type-1	*PSMB1*	-1.02
P25787	Proteasome subunit alpha type-2	*PSMA2*	-2.04
P05455	Lupus La protein	*SSB*	-1.65
P05388	60S acidic ribosomal protein P0	*RPLP0*	-1.46
P0DME0	Protein SETSIP	*SETSIP*	-1.49
P36871	Phosphoglucomutase-1	*PGM1*	-2.65
Q16836	Hydroxyacyl-coenzyme A dehydrogenase, mitochondrial	*HADH*	-1.55
Q8NHW5	60S acidic ribosomal protein P0-like	*RPLP0P6*	-1.43
Q02790	Peptidyl-prolyl cis-trans isomerase	*FKBP4*	-2.2
P35270	Sepiapterin reductase	*SPR*	-2.13
P25786	Proteasome subunit alpha type-1	*PSMA1*	-1.23
Q8N163	Cell cycle and apoptosis regulator protein 2	*CCAR2*	-2.38
P13796	Plastin-2	*LCP1*	-4.72
P27816	Microtubule-associated protein 4	*MAP4*	-1.21
P52788	Spermine synthase	*SMS*	-1.8
P61289	Proteasome activator complex subunit 3	*PSME3*	-3.47
P61077	Ubiquitin-conjugating enzyme E2 D3	*UBE2D3*	-2.15
Q14103	Heterogeneous nuclear ribonucleoprotein D0	*HNRNPD*	-1.64
Q99436	Proteasome subunit beta type-7	*PSMB7*	-3.68
P53675	Clathrin heavy chain 2	*CLTCL1*	-2.3
Q13310	Polyadenylate-binding protein 4	*PABPC4*	-1.39
P54577	Tyrosine-tRNA ligase, cytoplasmic	*YARS*	-2.15
Q15257	Serine/threonine-protein phosphatase 2A activator	*PTPA*	-3.18
O60506	Heterogeneous nuclear ribonucleoprotein Q	*SYNCRIP*	-1.69
O75915	PRA1 family protein 3	*ARL6IP5*	-3.35
O95373	Importin-7	*IPO7*	-2.37
P51665	26S proteasome non-ATPase regulatory subunit 7	*PSMD7*	-1.22
Q99729	Heterogeneous nuclear ribonucleoprotein A/B	*HNRNPAB*	-1.48
O15355	Protein phosphatase 1G	*PPM1G*	-3.2
P28072	Proteasome subunit beta type-6	*PSMB6*	-1.31
P29992	Guanine nucleotide-binding protein subunit alpha-11	*GNA11*	-2.59
Q9UBF2	Coatomer subunit gamma-2	*COPG2*	-2.84
O75821	Eukaryotic translation initiation factor 3 subunit G	*EIF3G*	-2.04
P61086	Ubiquitin-conjugating enzyme E2 K	*UBE2K*	-2.08
P09651	Heterogeneous nuclear ribonucleoprotein A1	*HNRNPA1*	-1.52
O14602	Eukaryotic translation initiation factor 1A	*EIF1AY*	-1.42
P61221	ATP-binding cassette sub-family E member 1	*ABCE1*	-2.82
Q13409	Cytoplasmic dynein 1 intermediate chain 2	*DYNC1I2*	-2.05
Q9UII2	ATPase inhibitor, mitochondrial	*ATP5IF1*	-3.86
Q969T9	WW domain-binding protein 2	*WBP2*	-1.7
Q9NR22	Protein arginine N-methyltransferase 8	*PRMT8*	-1.57
P07858	Cathepsin B	*CTSB*	-1.76

* *p* < 0.05.

**Table 2:** Differentially expressed nuclear proteins (*p* < 0.05) in the γT3-treated MDA-MB-231 cells compared with the untreated control.

**(A) Upregulated Nuclear Proteins**			
**Accession**	**Protein Description**	**Gene Symbol**	* Fold-change
P08133	Annexin A6	*ANXA6*	2.24
Q9NZM1	Myoferlin	*MYOF*	1.08
Q562R1	Beta-actin-like protein 2	*ACTBL2*	7.96
P16615	Sarcoplasmic/endoplasmic reticulum calcium ATPase 2	*ATP2A2*	1.2
P68363	Tubulin alpha-1B chain	*TUBA1B*	3.83
Q9BQE3	Tubulin alpha-1C chain	*TUBA1C*	2.37
Q71U36	Tubulin alpha-1A chain	*TUBA1A*	2.82
P09525	Annexin A4	*ANXA4*	2.31
P68366	Tubulin alpha-4A chain	*TUBA4A*	2.43
Q13748	Tubulin alpha-3C/D chain	*TUBA3C*	1.79
P46977	Dolichyl-diphosphooligosaccharide-protein glycosyltransferase subunit STT3A	*STT3A*	2.44
P35610	Sterol O-acyltransferase 1	*SOAT1*	2.79
O94905	Erlin-2	*ERLIN2*	1.25
Q9NY65	Tubulin alpha-8 chain	*TUBA8*	1.2
P50993	Sodium/potassium-transporting ATPase subunit alpha-2	*ATP1A2*	1.74
P13637	Sodium/potassium-transporting ATPase subunit alpha-3	*ATP1A3*	1.6
Q9Y6C9	Mitochondrial carrier homolog 2	*MTCH2*	1.33
Q9BWM7	Sideroflexin-3	*SFXN3*	1.17
Q9P035	Very-long-chain (3R)-3-hydroxyacyl-CoA dehydratase 3	*HACD3*	2.34
Q5JWF2	Guanine nucleotide-binding protein G(s) subunit alpha isoforms XLas	*GNAS*	2.31
Q14151	Scaffold attachment factor B2	*SAFB2*	3.14
Q9NZ01	Very-long-chain enoyl-CoA reductase	*TECR*	2.12
Q9HD45	Transmembrane 9 superfamily member 3	*TM9SF3*	2.38
Q9NQC3	Reticulon-4	*RTN4*	2.81
P14406	Cytochrome c oxidase subunit 7A2, mitochondrial	*COX7A2*	4.23
P18124	60S ribosomal protein L7	*RPL7*	3.08
P62917	60S ribosomal protein L8	*RPL8*	3.71
P46778	60S ribosomal protein L21	*RPL21*	3.6
P15153	Ras-related C3 botulinum toxin substrate 2	*RAC2*	2.78
P50151	Guanine nucleotide-binding protein G(I)/G(S)/G(O) subunit gamma-10	*GNG10*	1.52
P22090	40S ribosomal protein S4, Y isoform 1	*RPS4Y1*	2.29
P62913	60S ribosomal protein L11	*RPL11*	3.31
Q9UBM7	7-dehydrocholesterol reductase	*DHCR7*	2.51
Q9BQ39	ATP-dependent RNA helicase DDX50	*DDX50*	2.11
P35613	Basigin	*BSG*	1.28
Q9H299	SH3 domain-binding glutamic acid-rich-like protein 3	*SH3BGRL3*	1.62
P09669	Cytochrome c oxidase subunit 6C	*COX6C*	4.24
P46779	60S ribosomal protein L28	*RPL28*	1.39
P09496	Clathrin light chain A	*CLTA*	2.84
Q5TZA2	Rootletin	*CROCC*	3.44
Q13409	Cytoplasmic dynein 1 intermediate chain 2	*DYNC1I2*	1.47
Q13526	Peptidyl-prolyl cis-trans isomerase NIMA-interacting 1	*PIN1*	1.25
Q969 × 1	Protein lifeguard 3	*TMBIM1*	1.14
Q8TD47	40S ribosomal protein S4, Y isoform 2	*RPS4Y2*	2.3
O75964	ATP synthase subunit g, mitochondrial	*ATP5L*	2
P61313	60S ribosomal protein L15	*RPL15*	3.87
Q6NVV1	Putative 60S ribosomal protein L13a protein RPL13AP3	*RPL13AP3*	3.53
P14649	Myosin light chain 6B	*MYL6B*	2.51
Q96EP5	DAZ-associated protein 1	*DAZAP1*	3.1
Q71UM5	40S ribosomal protein S27-like	*RPS27L*	3.3
O95678	Keratin, type II cytoskeletal 75	*KRT75*	1.78
P24539	ATP synthase F(0) complex subunit B1, mitochondrial	*ATP5F1*	2.78
**(B) Down-Regulated Nuclear Proteins**			
**Accession**	**Protein Description**	**Gene Symbol**	* Fold-change
P31942	Heterogeneous nuclear ribonucleoprotein H3	*HNRNPH3*	-1.03
Q09161	Nuclear cap-binding protein subunit 1	*NCBP1*	-1.62
Q9NZN4	EH domain-containing protein 2	*EHD2*	-1.06
Q9Y262	Eukaryotic translation initiation factor 3 subunit L	*EIF3L*	-1.21
Q01105	Protein SET	*SET*	-1.36
P0DMV9	Heat shock 70 kDa protein 1B	*HSPA1B*	-1.89
Q9Y224	UPF0568 protein C14orf166	*RTRAF*	-1.09
O75390	Citrate synthase, mitochondrial	*CS*	-1.73
Q01844	RNA-binding protein EWS	*EWSR1*	-1.29
P68400	Casein kinase II subunit alpha	*CSNK2A1*	-1.23
P36776	Lon protease homolog, mitochondrial	*LONP1*	-1.44
Q13347	Eukaryotic translation initiation factor 3 subunit I	*EIF3I*	-1.32
Q96AE4	Far upstream element-binding protein 1	*FUBP1*	-1.29
P0DME0	Protein SETSIP	*SETSIP*	-1.45
Q9UQE7	Structural maintenance of chromosomes protein 3	*SMC3*	-1.11
P63279	SUMO-conjugating enzyme UBC9	*UBE2I*	-2.08
Q96FQ6	Protein S100-A16	*S100A16*	-1.06
P25205	DNA replication licensing factor MCM3	*MCM3*	-1.51
Q03518	Antigen peptide transporter 1	*TAP1*	-1.81
Q8IX12	Cell division cycle and apoptosis regulator protein 1	*CCAR1*	-1.38
P53701	Cytochrome c-type heme lyase	*HCCS*	-1.82
P82930	28S ribosomal protein S34, mitochondrial	*MRPS34*	-1.28
O60814	Histone H2B type 1-K	*HIST1H2BK*	-1.88
Q01085	Nucleolysin TIAR	*TIAL1*	-1.89
O75923	Dysferlin	*DYSF*	-1.23
Q96ND0	Protein FAM210A	*FAM210A*	-3.82
M2P21980	Protein-glutamine gamma-glutamyltransferase 2	*TGM2*	-1.54

* *p* < 0.05.

**Table 3:** List of the unique proteins in the union and intersections between the cytoplasmic and nuclear protein compartment of MDA-MB-231 cells in response to γT3 treatment.

Compartment	Total	Elements
Cytoplasmic and Nuclear	7	ATP1A3, TUBA1C, LONP1, DYNC1I2, SET, RAC2 and SETSIP
Cytoplasmic Proteins	104	SMS, HSPD1, PSMA2, PSMB1, MAP4, CCAR2, FH, PRMT1, UBE2V2, DBI, ACTA2, CLTCL1, ATP1A4, ECHS1, CD151, PTTG1IP, CD9, PSMB6, HSD17B10, PPIAL4A, COPG1, RAC1, UBE2NL, ALCAM, ACTG2, EIF1AY, CYCS, PABPC4, SSB, HADH, ARL6IP5, CTSB, UBE2K, PRDX3, PSME3, COPG2, XRCC6, DYNLL1, RAB25, GANAB, PCBP3, HNRNPD, RPLP0, PSME2, SEC22B, HNRNPAB, YARS, COTL1, RPLP0P6, GSN, SYNCRIP, MYH14, TUFM, PTPA, IPO7, TPD52L2, GNA11, RAB11A, CD59, PRMT8, GNB2, PGM1, ACTC1, ATP1B3, RPLP2, PSAP, SPR, PCNA, MYDGF, ABCE1, HLA-A, WBP2, LCP1, SCAMP4, PSMA1, NCL, PC, HNRNPA1, SCAMP2, VAMP3, ATP5IF1, FKBP1A, CTTN, EIF3G, PSMB7, SHMT1, PPM1G, RAB1A, TRIM28, UBE2D3, SHMT2, PSMD7, SDHA, FKBP4, RAB2B, PCBP2, NPC2, RAB6A, CD97, PSMD2, CBR1, ALDH2, ATP1B1 and OAT
Nuclear Proteins	72	RPL13AP3, PIN1, CCAR1, STT3A, MCM3, EIF3I, RTN4, ACTBL2, ATP5F1, MYL6B, TM9SF3, ANXA6, TUBA4A, EWSR1, DAZAP1, ERLIN2, RTRAF, RPL28, RPL7, MYOF, SH3BGRL3, DHCR7, GNG10, TMBIM1, RPS27L, COX6C, ANXA4, TUBA3C, RPS4Y1, ATP2A2, MTCH2, TUBA1A, S100A16, HSPA1B, TAP1, NCBP1, COX7A2, CS, ATP5L, ATP1A2, SOAT1, CROCC, CSNK2A1, TGM2, HNRNPH3, TECR, RPL21, HACD3, CLTA, SMC3, RPL11, SFXN3, RPL8, DDX50, MRPS34, EHD2, GNAS, EIF3L, DYSF, TUBA8, FUBP1, HIST1H2BK, FAM210A, TUBA1B, KRT75, HCCS, UBE2I, BSG, SAFB2, TIAL1, RPS4Y2 and RPL15
